# Cardiac Amyloidosis: Updates in Imaging

**DOI:** 10.1007/s11886-019-1180-2

**Published:** 2019-08-02

**Authors:** Liza Chacko, Raffaele Martone, Francesco Cappelli, Marianna Fontana

**Affiliations:** 1 National Amyloidosis Centre, University College London, Royal Free Campus, Rowland Hill Street, NW3 2PF, London, UK; 2 Tuscan Regional Amyloid Center, Careggi University Hospital, Florence, Italy

**Keywords:** Cardiac amyloidosis, Magnetic resonance imaging, Cardiomyopathy, Immunoglobulin light chain, Transthyretin, Echocardiography

## Abstract

**Purpose of Review:**

We summarize key features pertaining to the two most commonly encountered types of cardiac amyloidosis (CA), monoclonal immunoglobulin light chain (AL) and transthyretin type (ATTR), expanding upon the clinical application and utility of various imaging techniques in diagnosing CA.

**Recent Findings:**

Advances in imaging have led to earlier identification, improved diagnosis of CA and higher discriminatory power to differentiate CA from other hypertrophic phenocopies. The application of cardiac magnetic resonance imaging (CMR) has led to a deeper understanding of underlying pathophysiological processes in CA, owing largely to its intrinsic tissue characterization properties. The widespread adoption of bone scintigraphy algorithms has reduced the need for cardiac biopsy and improved diagnostic confidence in ATTR CA.

**Summary:**

As new treatments for CA are rapidly developing, there will be even greater reliance on imaging, as the requirement to diagnose disease earlier, monitor response and amend treatment strategies accordingly intensifies.

## Introduction

The systemic amyloidoses comprise a heterogenous group of diseases that are characterized by the extracellular deposition of proteins that misfold, aggregate and deposit as amyloid fibrils causing disease when accumulation is sufficient to disrupt the structure and integrity of affected organs [1]. Although up to thirty different proteins can deposit as amyloid, defined histologically by staining with Congo Red to produce characteristic apple green birefringence under cross polarized light, studies have demonstrated that amyloid fibrils share a common core structure of highly ordered, abnormal anti parallel beta strands that form sheets, properties of which include relative stability and resistance to proteolysis [1, 2]. Cardiac amyloidosis (CA) occurs when amyloid fibrils deposit within the myocardial extracellular space, causing interruption and distortion of myocardial contractile elements, stiffness of the ventricles and systolic and diastolic dysfunction. Although amyloid is often a multi-organ disease, cardiac involvement is the leading cause of morbidity and mortality [3, 4]. The majority of cases of CA can be attributed to two precursor proteins: the monoclonal immunoglobulin light chain (AL) protein type which is produced by an abnormal clonal proliferation of plasma cells, and the transthyretin (ATTR) protein type which is liver derived and normally involved in the transport of thyroxine and retinol binding protein. [5, 6] Wider awareness of CA as an underdiagnosed cause of restrictive cardiomyopathy, in conjunction with advances in imaging modalities including bone scintigraphy, and cardiac magnetic resonance imaging (CMR) over the past decade have helped to transform the profile of CA allowing for crucial earlier diagnosis, better understanding of underlying disease processes, and an ability to track disease in response to treatment. In the following review article, we summarize key features pertaining to the two most common types of cardiac amyloidosis, with predominant focus on the clinical application and utility of imaging modalities in diagnosing CA and the influence of imaging towards treatment.

## Overview of AL and ATTR Amyloidosis

Historically, systemic immunoglobulin amyloidosis (AL) was considered to be the most common type of amyloidosis with an estimated prevalence of 8 to 12 per million person years. [7, 8] The associated clinical phenotype and symptomatology are diverse, reflecting the potential for amyloid infiltration to affect multiple organs. Diagnostic delays often occur due to the non-specific nature of symptoms including but not limited to fatigue, dyspnoea and weight loss. More specific clinical signs such as macroglossia and periorbital bruising are essentially pathognomonic but occur only in up to one third of cases [2]. Cardiac involvement affects up to 80% of patients with AL CA [9], and patients present with heart failure symptoms. Because the disease affects all cardiac chambers, biventricular dysfunction is usually present, although the most frequent presenting feature is severe right-sided heart failure. Despite best medical treatment, the prognosis of AL CA remains poor [10].

ATTR CA is classified into the hereditary form (hATTR) or non-hereditary form which is known as wild type ATTR (wtATTR) based on the type of transthyretin protein. [11] The diagnosis of wtATTR has risen considerably in recent years, with estimates of 13–16% prevalence in older patients presenting with heart failure with preserved ejection fraction (EF) [12, 13]. Wild type ATTR CA (formerly known as senile systemic CA) has a male predominance, and although presents with a predominant cardiac phenotype and restrictive cardiomyopathy, it has often been associated with lumbar canal stenosis, carpal tunnel syndrome and/or tendinopathy. [14–17] This is in contrast to the clinical phenotype of hATTR amyloidosis which presents at a younger age with a variable clinical presentation usually comprising a mixed phenotype with peripheral neuropathy, autonomic neuropathy and/or cardiomyopathy [18]. There are over 120 causative TTR mutations [19], the most common being V122I which is present in up to 3.4% of US African Americans, the clinical presentation and onset of which closely mimics ATTRwt. [20] It is estimated that approximately 2 million people in the US are carriers of this variant, and at risk of developing CA. Patients with nervous system involvement often experience disabling neurological symptoms however, similar to AL amyloidosis, cardiac involvement in ATTR has the most important impact on prognosis carrying a median survival of 4–5 years [21].

## Imaging

### Echocardiography

**Fig. 1:**
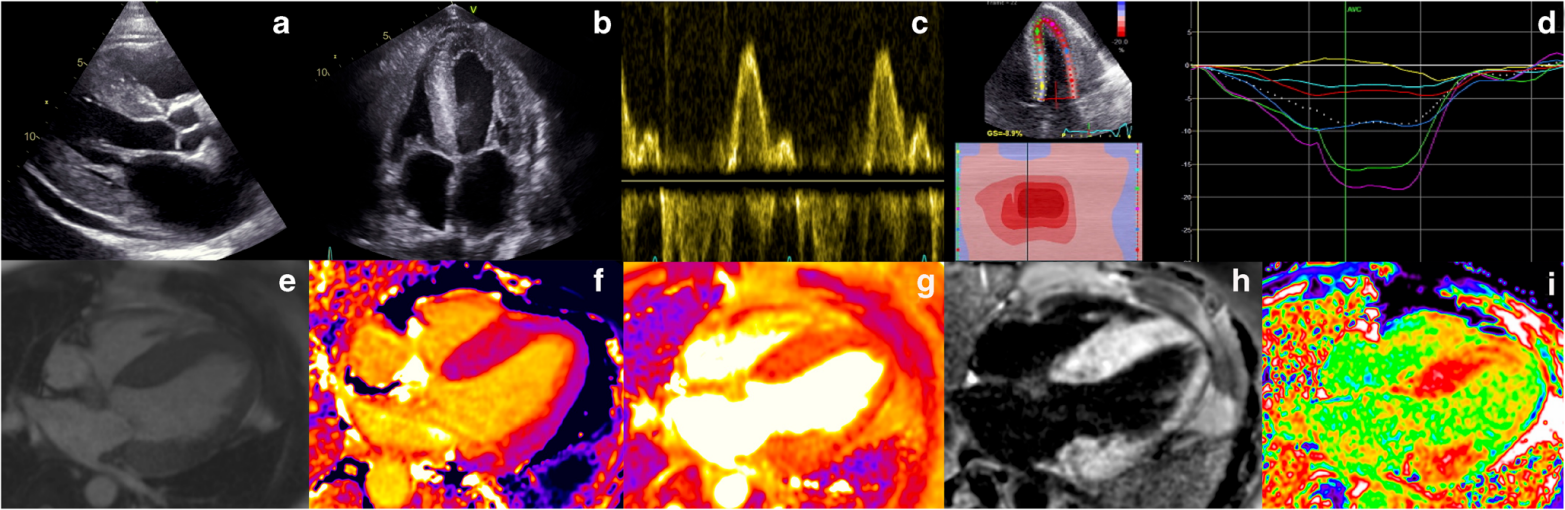
**Top Panel:** Echocardiography findings in a patient with advanced cardiac amyloidosis. **(a)** Parasternal long axis view and **(b)** four chamber view showing concentric left ventricular hypertrophy **(c)** pulse wave Doppler showing restrictive left ventricular inflow pattern **(d)** strain pattern characteristic of an infiltrative process. **Bottom Panel:** CMR findings in a patient with advanced cardiac amyloidosis. **(e)** Four chamber steady state free precession cine demonstrating left ventricular hypertrophy **(f)** corresponding native T1 map showing a T1 value of 1150 ms in the basal inferoseptum **(g)** corresponding T2 map showing a T2 value of 54 ms in the basal inferoseptum, within normal limits **(h)** corresponding phase sensitive inversion recovery reconstruction showing transmural late gadolinium enhancement **(i)** corresponding extracellular volume map showing elevated value of 0.70

Echocardiography is the most accessible and first line imaging tool in the approach towards assessing patients with cardiomyopathy. The amyloid phenotype is one of characteristic biventricular wall thickening with small, non-dilated ventricles and left ventricular (LV) wall thickness typically greater than 12 mm (Fig. 1). There is a tendency towards a symmetrical increase in LV wall thickness in AL CA, while ATTR CA more often demonstrates an asymmetrical pattern. [22, 23•] In the latter, the morphology of the septum may be sigmoid (seen in 70%) or demonstrate inversion of the septal curvature (seen in 30%). [22] Although patients with ATTR CA typically have higher LV and RV mass at diagnosis, which may in fact reflect earlier clinical presentation in patients with AL CA [24], LV mass in isolation is unsuited to differentiate between the types. Well described but non-specific findings of CA include a thickened and sparkling appearance of the valves and interatrial septum, as well as a ‘speckled’ appearance of the myocardium. Pericardial and pleural effusions are also relatively common findings, especially in AL amyloidosis.

Amyloid infiltration in the extracellular space leads to ventricular stiffening, impaired relaxation and biventricular diastolic dysfunction, which in combination with direct atrial amyloid infiltration can lead to atrial dilatation, blood stasis and a higher risk of thrombus formation [25–28]. Although CA is traditionally categorized as a cause of ‘heart failure with preserved EF’, this under-represents the extent of involvement of both systolic and diastolic dysfunction. Ejection fraction, which is a widely relied upon measure of ventricular function is not a reliable indicator of systolic function in CA as EF reflects radial contraction which is often preserved until end stage disease. Longitudinal function is typically affected earlier than radial contraction and indices of longitudinal function can be used as early disease markers. This was initially demonstrated measuring the systolic excursion of the mitral annulus evaluated by Tissue Doppler imaging (TDI) or from M-mode derived mitral annular plane systolic excursion (MAPSE) [29, 30], and later on with strain imaging. Longitudinal strain (LS) measurement by tissue Doppler and echocardiographic speckle tracking are proving to be valued tools in diagnosing CA, as well as differentiating CA from other hypertrophic phenocopies [3]. Strain demonstrates not only reduction in longitudinal contraction, but also reduction in LS that affects predominantly the basal segments with sparing of the apical segments. This is a highly characteristic feature of CA, which gives rise to the typical appearance of a ‘bull’s eye pattern’ with strain values that are reduced on the side and preserved in the centre of the plot (Fig. 1) [3, 31]. The extent of apical sparing can be quantified using relative ratio between apical and basal LV regional strain, which is also associated with poorer prognosis [32].

In CA patients diastolic function is almost invariably impaired and the degree of impairment ranges from impaired relaxation to restrictive filling patterns [33]. Parameters of diastolic dysfunction can also be used as early disease markers, with TDI of the mitral annulus often being less than 6 cm/s (Fig. 1) [34].

### Cardiovascular Magnetic Resonance

As a now well-established imaging modality that is instrumental in the approach towards cardiomyopathies, CMR provides unparalled accuracy on cardiac morphology.

and informs upon tissue composition through its intrinsic capacity to define myocardial tissue characterization. The deposition of amyloid fibrils in the extracellular myocardial space leads to expansion of the extracellular volume, which is well visualized by the administration of gadolinium-based contrast agents, referred to as ‘late gadolinium enhancement’ (LGE). Gadolinium accumulates passively in gaps between myocardial cells giving rise to the appearance of diffuse subendocardial or transmural LGE in CA, in the presence of abnormal myocardial and blood pool gadolinium characteristics, a phenomenon that was recognized over 10 years ago. [35] LGE differentiates normal from abnormal myocardium, based on the assumption that there are remote regions of normal myocardium. However, this may not exist in diffuse infiltrative diseases such as CA, exposing an area for potential operator error whereby the operator may erroneously null the abnormal and not normal myocardium, carrying a risk of reporting ‘false negative’ examinations or ‘mirror images’ of the true pattern. [36] The LGE technique has matured over the years leading to the wide adoption of ‘phase sensitive image reconstruction’ (PSIR) which is a more robust and reliable technique than magnitude reconstruction with the primary advantage that it largely overrides the dependence on operator determined optimal null point and related errors [36]. With the PSIR LGE approach, 3 patterns of LGE have been recognized; none, sub-endocardial and transmural, and transmurality of LGE shows good correlation with the degree of myocardial infiltration. (Fig. 1) [36] An important drawback of LGE is that gadolinium-based contrast agents (GBCA) have been associated with nephrogenic systemic fibrosis (NSF), a serious and potentially fatal condition. Whilst the risk of developing NSF is strongly related to baseline renal function (being the highest when eGFR <30 mL/min), the underlying chemical structure of the contrast agent also plays an important role in determining risk. Recent guidelines from the American College of Radiology recommend the preferential use of Group II agents in patients at risk of NSF if clinically indicated, emphasizing the requirement for a balanced assessment of the risks of administrating GBCA against the risks of not performing a contrast scan. [37] Whilst the initial understanding was that the gadolinium ion remained in a chelated state after intravenous administration, multiple studies have demonstrated evidence of tissue retention, even in patients with normal renal function [38] including reports of involvement in neural tissue (dentate nucleus, thalamus, pons, and globus pallidus) [39–41] and bone tissue, [42] clinical implications of which are not fully understood. A further limitation of LGE is that it cannot be used to track changes in disease status over time due its non-quantitative nature.

These limitations can be overcome by the use of T1 mapping which directly measures an intrinsic signal from the myocardium, the longitudinal relaxation time, in a pixel wise manner. (Fig. 1) Native (pre-contrast) myocardial T1 tracks cardiac amyloid infiltration, markers of systolic and diastolic dysfunction and disease severity [43].

Important advantages of native myocardial T1 are its diagnostic accuracy for detecting CA in both AL and ATTR types and its role as an early disease marker, frequently found to be elevated prior to the onset of disease features such as LV hypertrophy or LGE [43, 44].

Native T1 is a composite signal, from both the extra and intracellular space. Following the administration of gadolinium contrast agents, from the ratio of pre and post contrast T1 and haematocrit, the signal from the extracellular space can be isolated with the measurement of the extracellular volume (ECV).

ECV is the first non-invasive method for quantifying the cardiac amyloid burden, and several studies have shown correlation with markers of disease severity in both types of CA. [22, 45] The ECV is globally elevated, often with values >40% and higher in ATTR than AL CA. (Fig. 1) Important benefits of ECV measurement in CA include its unique ability to measure the continuum of amyloid infiltration, to track markers of disease activity such as cardiac function, blood biomarkers and functional performance, to act as an early disease marker and to uniquely track changes over time. [45] For example, almost half of the patients in a studied cohort who achieved a good clonal response to chemotherapy in AL amyloidosis demonstrated evidence of regression of cardiac amyloid on ECV [46].

In conjunction with detailed morphological and functional assessments, tissue characterization by CMR provide a wholesome understanding of the multiple disease processes that exist within CA, transcending the concept of CA as a disease of solely infiltration. T2 relaxation time is a time constant representing the decay of transverse magnetization and detects oedema in various pathologies including but not limited to acute myocardial infarction, myocarditis, and Takotsubo cardiomyopathy. (Fig. 1) [47] Recently, T2 mapping in CA has added significantly to our understanding of CA as a heterogenous condition comprising multiple disease processes by demonstrating that T2 levels were higher in a cohort of patients with untreated AL CA compared with treated AL and ATTR CA, thereby showing oedema to have both important pathophysiological and prognostic roles [48•].

### Bone Scintigraphy

It has been recognized since the 1980’s that patients affected by CA were incidentally observed to demonstrate uptake of certain ^99m^Tc-phosphate derivative, following which began the application of bone scintigraphy in CA. Although the basis for localisation of these agents to CA remains unclear, the technique is sensitive for diagnosing ATTR CA. In 2005, a small yet seminal paper demonstrated the strong diagnostic potential of ^99m^ Technetium-labelled 3,3-dicarboxypropane-2, 1-diphosphonate (^99m^Tc-DPD) in identifying ATTR CA. [49] Further studies confirmed this finding, as well as the utility of other bone tracers in identifying ATTR CA.

These seminal findings were recently reinforced by results from a large multicentre trial which demonstrated the ability of bone scintigraphy to diagnose cardiac ATTR CA reliably without the need for histology, the diagnostic algorithm from which has been widely recognized and adopted in clinical practice. [50] Briefly summarized, in patients in whom free light chains are absent in the blood and urine, once CA is suspected and ^99m^Tc-PYP (^99m^ Technetium labelled pyrophosphate /^99m^Tc-DPD/^99m^Tc-HMDP (^99m^ Technetium labelled hydroxymethylene diphosphonate) is negative, CA is very unlikely. If the ^99m^Tc-PYP/^99m^Tc-DPD/^99m^Tc-HMDP cardiac scan is positive for either grade 2 or 3, and there is no evidence of free light chains in the blood and/or urine, ATTR CA can be diagnosed without a biopsy (specificity and positive predictive value >98%). However, if patients have evidence of a plasma cell dyscrasia, further definitive tests such as biopsies are still required as part of the algorithm as the presence of low grade uptake on a ^99m^Tc-PYP/^99m^Tc-DPD/^99m^Tc-HMDP cardiac scan does not confer 100% specificity for ATTR CA, and mild cardiac localisation may be seen in certain patients with advanced AL CA, cardiac Apo1 and amyloid A amyloidosis [50–52].

An intriguing - yet not fully explored - field is the potential of bone-tracers for the assessment of extra-cardiac involvement in systemic amyloidosis. A typical pattern of muscle and soft-tissue uptake of ^99m^Tc-DPD has been previously reported [52] and amyloid tissue infiltration has been later demonstrated by soft tissue biopsy in a larger series of positive patients [53]. Lung uptake may be found at ^99m^Tc-HMDP scintigraphy [54], with high selectivity for ATTR. The clinical implications of these findings are not completely understood. Notably, extracardiac uptake appears to be tracer-specific, as Sperry et al. could not find any relevant skeletal muscle uptake at ^99m^Tc-PYP scintigraphy [55].

#### Positron Emission Tomography

Positron emission tomography (PET) imaging offers high spatial resolution, and may facilitate absolute quantification of cardiac and extracardiac amyloid burden [56]. PET amyloid binding radiotracers that have been studied in patients with AL and ATTR CA include 11-C-Pittsburgh compound B (^11^C PiB) [57, 58], ^18^F-florbetapir [56, 59], and ^18^F-florbetaben [60]. In these pilot studies, high cardiac radiotracer uptake was consistently reported in patients with CA compared to controls, including studies that used hypertensive heart disease as a control [60]. Although results from the aforementioned studies are encouraging, further evaluation of PET radiotracers is warranted prior to their incorporation into clinical practice.

### Diagnosis

Several factors contribute to the under diagnosis of CA. These include phenotypic heterogeneity, low index of clinical suspicion in the presence of overlap with more commonly seen phenocopies (hypertension, chronic renal failure, hypertrophic cardiomyopathy, aortic stenosis), a historical lack of non-invasive diagnostic tests, and limited understanding of the available treatment options.

**Fig. 2:**
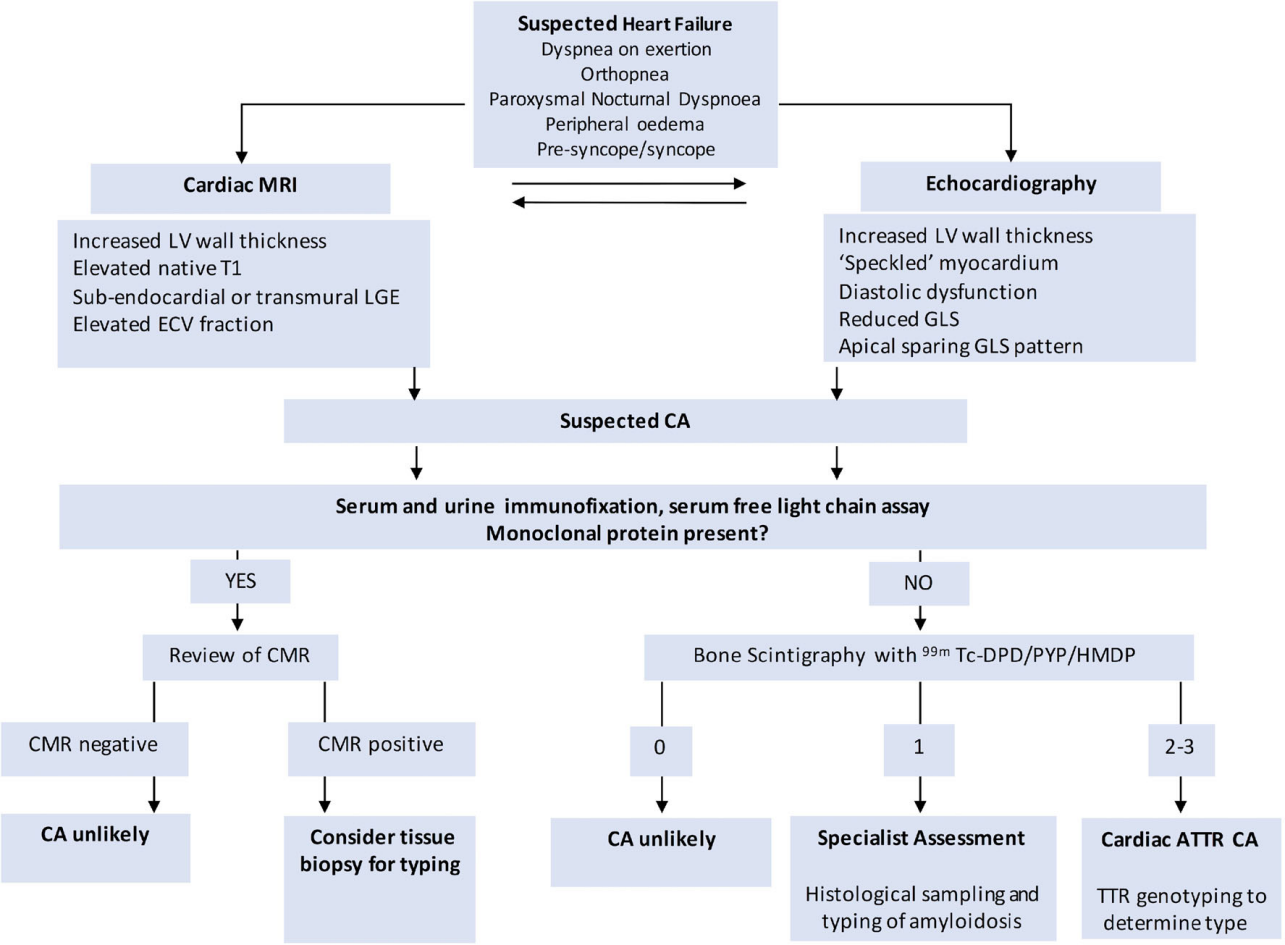
Schematic diagram representing the diagnostic pathway for cardiac amyloidosis

Current non-invasive diagnostic algorithms follow an integrated and multimodality approach towards diagnosing CA. (Fig. 2) Important factors to consider include the presence or absence of plasma cell dyscrasia, suggestive features on echocardiography and CMR imaging and as appropriate, histological samples and bone scintigraphy (Fig. 2).

Two frequently faced scenarios that raise a diagnostic challenge include [1] distinguishing CA from other more commonly seen hypertrophic phenotypes such as hypertensive heart disease, aortic stenosis, hypertrophic cardiomyopathy and restrictive cardiomyopathy; [2] Assessing for the CA on the background of known systemic AL or ATTR in specific scenarios such as in patients with renal AL where confounding comorbidities and often the inability to use gadolinium based contrast agents makes the diagnosis more challenging or in patients with an ATTR related polyneuropathy.

Echocardiography is the most commonly performed first line imaging modality for patients presenting with signs and symptoms of heart failure. Whilst the majority of echocardiographic findings in CA are non-specific, these can be highly suggestive, and alter the pre-test probability.

### Diagnosing Cardiac Amyloidosis in the Hypertrophic Phenotype

Once echocardiography has raised the suspicion of CA, CMR should be considered if both AL and ATTR or another underlying cause of myocardial hypertrophy (hypertension, hypertrophic cardiomyopathy, Anderson Fabry) are in the differentials. Following a positive CMR, ^99m^Tc-PYP/^99m^Tc-DPD /^99m^Tc-HMDP scan in combination with the assessment of free light chains in the blood and urine should be performed to distinguish between AL and ATTR amyloidosis.

### Diagnosing Cardiac Amyloidosis Patients with Known Systemic AL or ATTR Amyloidosis

In patients with systemic AL, CMR should be considered the imaging of choice to confirm cardiac involvement or detect early disease. CMR has been shown to have high sensitivity and specificity for AL CA [61], picking up early disease even when there is insufficient cardiac infiltration for the diagnosis to be made on echocardiography. CMR or bone scintigraphy should be considered in patients with ATTR polyneuropathy or ATTR mutation carriers, but further studies are needed in these patient populations.

### Prognosis

Blood biomarkers play a primary role in the stratification of AL and ATTR CA. The Mayo Classification of AL CA uses NT-proBNP and troponin measurements to categorize patients into Grade 0 (both values below threshold), Grade 1 (either value above threshold), and Grade 2 (both values above threshold) providing a valuable prognostic tool as an adjunct to other investigations. [62] In ATTR CA two different prognostic classification have been developed, one based on troponin and N-terminal pro–B-type natriuretic peptide (NT-proBNP), and one based on NT-proBNP and eGFR classifying as such: Stage 1 (both values below threshold), Stage 2 (either value above threshold), and Stage 3 (both values above threshold) [63, 64].

There are several structural and functional parameters seen on echocardiography and CMR that correlate with prognosis in both AL and ATTR amyloidosis, however on multivariable assessment, tricuspid annular plane systolic excursion (TAPSE) and stroke volume (SV) seem to be the strongest markers of prognosis in patients with CA [65]. Whilst RV failure is well documented independent predictor of prognosis in patients with primary left heart failure, the principal reason behind the prognostic importance of TAPSE in CA is likely to be direct sub-endocardial infiltration rather than RV dysfunction secondary to LV impairment. The prognostic role of SV in CA [65] is in keeping with the expected features of a restrictive cardiomyopathy characterized by low stroke volume despite relative preserved EF. A recent prospective registry of patients with both AL CA and wild type ATTR CA found that patients with AL CA, LV global longitudinal strain was predictive for outcome even after multivariable adjustment, whilst with wild type ATTR CA, RV free wall strain was the most powerful predictor of cardiac outcome [66].

MRI parameters that have a prognostic role include the transmurality of LGE, ECV,T1 and T2 in AL CA [36, 48•, 67], and ECV in ATTR. [22] For ^99m^Tc-PYP/^99m^Tc-DPD /^99m^Tc-HMDP scans, grade 1 carried a more favourable prognosis than grade 2 and 3 [53].

### Current Therapies, and the Future

An important area of expansion is in the domain of treatment in both AL and ATTR CA. In AL amyloidosis, treatment strategies are aimed at rapidly suppressing the production of amyloidogenic light chains, central to which is cytotoxic chemotherapy [2]. Although patients are assessed early for eligibility for stem cell transplant, the majority are considered ineligible owing to age, renal function, and advanced cardiac involvement [68]. Treatment options are tailored as per individual patient profile and risk based on performance status, experience which stems from treatments in multiple myeloma. Chemotherapeutic agents include combinations of bortezomib, melphalan, dexamethasone, cyclophosphamide, lenalidomide and other agents. Daratumumab which is an anti-plasma cell therapy for the treatment of relapsed multiple myeloma shows promising activity in patients with AL amyloidosis. [68] Other promising areas of development include monoclonal antibody therapy that aim to target existing amyloid deposits [69•].

There has been a significant expansion in pharmacotherapy directed at ATTR CA, and the approach towards treatment involves reducing or eliminating the production of transthyretin, disrupting the already deposited amyloid fibrils or stabilizing the protein. [70] Inotersen, a 2′-O-methoxyethyl–modified antisense oligonucleotide that inhibits hepatic production of TTR has been found in a randomized controlled trial of patients with ATTRm with polyneuropathy to improve quality of life, and modify neurological disease [71]. In a landmark study, in patients with ATTR CA, tafamidis, a TTR stabilizer, was shown to be associated with reductions in all-cause mortality and cardiovascular-related hospitalizations. There was also benefit seen in functional capacity and quality of life as compared to placebo [72]. In the cardiac sub population of the drug trial patisaran, an RNA interference agent, there was reduced echocardiographic wall thickness, global longitudinal strain, NT-proBNP compared with placebo at eighteen months [73].

## Conclusion

With rapid advances in treatment strategies, the fundamental goal of imaging is to focus on earlier diagnosis, treatment, and subsequent improvement in patient quality of life and survival. Imaging holds a key role in delineating and understanding the various disease mechanisms involved in CA. This richer understanding will continue to transform the profile of CA, allowing for treatment strategies to be tailored to patient disease characteristics, and for response to treatment to be tracked effectively, ultimately ending in a more streamlined and satisfactory patient experience.

## Abbreviations

CA: Cardiac amyloidosis
AL: Monoclonal immunoglobulin light chain
ATTR: Transthyretin
CMR: Cardiovascular magnetic resonance
hATTR: Hereditary ATTR
wtATTR: Wild type ATTR
EF: Ejection fraction
LV: Left ventricular
TDI: Tissue doppler imaging
MAPSE: Mitral annular plane systolic excursion
LS: Longitudinal strain
LGE: Late gadolinium enhancement
PSIR: Phase sensitive image reconstruction
GBCA: Gadolinium-based contrast agents
ECV: Extracellular volume
^99m^Tc-DPD: 99 m Technetium labelled 3,3-diphosphono-1,2-propanodicarboxylic acid
^99m^Tc-PYP: 99 m Technetium labelled pyrophosphate
^99m^Tc-HMDP: 99 m Technetium labelled hydroxymethylene diphosphonate
PET: Positron emission tomography
^11^C PiB: 11-CPittsburgh compound B
NT-proBNP: N-Terminal pro–B-type natriuretic peptide
TAPSE: Tricuspid annular plane systolic excursion
SV: Stroke volume
